# The impact of a fasting mimicking diet on the metabolic health of a prospective cohort of patients with prostate cancer: a pilot implementation study

**DOI:** 10.1038/s41391-022-00528-3

**Published:** 2022-03-21

**Authors:** V. Fay-Watt, S. O’Connor, D. Roshan, A. C. Romeo, V. D. Longo, F. J. Sullivan

**Affiliations:** 1 School of Medicine, National University of Ireland, Galway, Ireland; 2 Department of Radiation Oncology, Galway Clinic, Doughiske, Galway, Ireland; 3 School of Mathematical and Statistical Sciences, National University of Ireland, Galway, Ireland; 4 CURAM, SFI Research Centre for Medical Devices, National University of Ireland, Galway, Ireland; 5 Department of Internal Medicine and Medical Specialties, IRCCS Ospedale Policlinico San Martino, Genova, Italy; 6 IFOM, FIRC Institute of Molecular Oncology, Via Adamello 16, 20139 Milano, Italy; 7 Longevity Institute, School of Gerontology, Department of Biological Sciences, University of Southern California, 3715 McClintock Avenue, Los Angeles, CA 90089-0191 USA; 8 Department of Radiation Oncology, Galway Clinic, Prostate Cancer Institute, National University of Ireland, Galway, Ireland

**Keywords:** Cancer therapy, Cancer prevention, Prostate cancer, Prostate cancer, Cancer metabolism

## Abstract

**Background:**

This pilot prospective study investigated the effect of a periodic fasting mimicking diet (FMD) on metabolic health factors in patients with Prostate Cancer (PC). There is a well-documented association between PC and metabolic health. Impaired metabolic health is a significant risk factor for the development of PC, and a metabolic syndrome can be induced by hormonal therapies commonly required for its management. (ClinicalTrials.gov Identifier: NCT04292041).

**Methods:**

We introduced a periodic 4-day FMD -low in calories, sugars, and proteins but high in unsaturated fats -to a cohort of PC patients and features of metabolic syndrome. 29/35 patients completed 3-monthly cycles of the 4-consecutive day packaged FMD. We compared the subjects’ baseline weight, abdominal circumference (AC), blood pressure (BP) and selected laboratory results to the same measurements 3-months after completing the FMD cycles.

**Results:**

Several important metabolic factors showed improvements post-intervention. On average patients’ weights dropped by 3.79 kg (95% CI: −5.61, −1.97, *p* = 0.0002). AC was reduced on average by 4.57 cm, (95% CI: −2.27, −6.87, *p* = 0.0003). There was also a decrease in systolic and diastolic BP by 9.52 mmHg (95% CI: −16.16, −2.88, *p* = 0.0066) and 4.48 mmHg (95% CI: −8.85, −0.43, *p* = 0.0316) respectively. A sub-analysis indicates that FMD had more relevant effects in ‘at-risk’ patients than those with normal values of risk factors for metabolic syndrome. For example, subjects with baseline levels of systolic BP > 130 mmHg experienced a greater reduction in BP(−16.04 mmHg, *p* = 0.0001) than those with baseline systolic BP < 130 mmHg (−0.78 mmHg, *p* = 0.89).

**Conclusions:**

The FMD cycles were safely introduced to this small cohort of PC patients with little or no observed toxicity, and a high overall compliance of 83%. Analysis of the metabolic variables showed an overall decrease in weight, AC, and BP. Larger clinical trials focused on metabolic risk factors, PC quality of life and progression free survival are needed to assess the effect of the FMD on prostate cancer patients.

## Introduction

Prostate cancer (PC) is the second most common cancer in males. Its high incidence, disease burden and fatality makes this cancer a major global health problem [1]. PC is a hormone sensitive secondary sex-organ cancer, and patients are particularly susceptible to the metabolic syndrome due to the cancer’s well-known association with weight gain, and co-morbidities such as diabetes [2]. Androgen deprivation therapy (ADT), using drugs designed to block the effects of testosterone and related androgens, is an important strategy in the management of prostate cancer [3]. These drugs are known to produce significant metabolic side effects. Consequently, PC patients develop many features of a metabolic syndrome as an iatrogenic effect of these drugs. This makes PC patients an ideal study group in which to evaluate the effect of nutritional and other lifestyle interventions, to improve quality of life, and potentially improve overall mortality in this condition.

Independent of ADT, obesity has well-established links with PC including being a risk factor for aggressive disease, and increased cause-specific mortality [4]. Recent literature reports that in men, a weight gain more than 5% of the body weight after prostate cancer diagnosis is associated with a 65% increased risk of dying of prostate cancer and 27% higher risk of all-cause mortality [5, 6]. A lifestyle modification leading to weight loss could delay or prevent disease progression, as well as improve quality of life [7].

Intermittent fasting has been advocated as an effective method of weight loss in chronic illnesses such as Type II diabetes but recommending any type of “fasting” to a cancer patient is considered controversial in the oncology community. However, animal and some initial human studies indicate that fasting for as little as 3–5 days done periodically can be very effective in treating a variety of cancers when combined with standard of care [8]. The Fasting mimicking Diet (FMD) is a form of periodic fasting, low in calories, sugars, and protein but high in unsaturated fats. Rodent studies established that a periodic 4-day FMD administered twice per month, alternating with a normal diet, cause significant weight/visceral-fat loss, as well as a reduction in tumor incidence by 45% and delay tumor development [9]. Three cycles of a FMD, were shown to be effective in reducing body weight, trunk and total body fat, blood pressure, and IGF-1 in comparison to a normal diet in a randomized phase 2 trial enrolling one-hundred participants, without a diagnosed medical condition [10]. Thus, there is a sound pre-clinical as well as clinical rationale for the use of FMD in PC. This study examined whether three monthly-cycles of FMD would be accepted, tolerated and safely completed by PC patients. Furthermore, it assessed its effect on metabolic risk factors associated with poor prognosis and features of a metabolic syndrome. We tested this in a small opportunistic trial including a representative cohort of men with prostate cancer, at various stages of their illness. This represents the first publication on the use of FMD in PC patients.

## Materials and methods

**Fig. 1:**
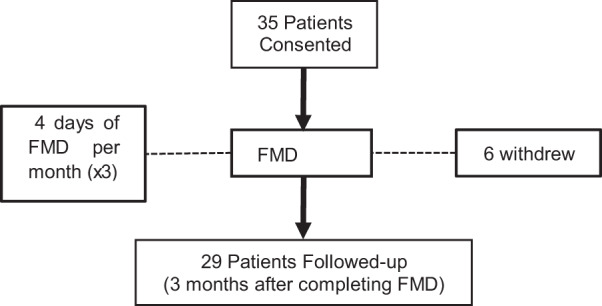
Study design. 35 patients were enrolled in the study, 6 patients withdrew during various FMD cycles. 29 Patients completed 3 cycles of a 4 -day FMD per month. Participants' weight, abdominal circumference, blood pressure and laboratory markers were evaluated 3 months after completion of the FMD cycles.

The cohort was the result of an opportunistic selection of a prospective group of men, treated in a community oncology setting. Subjects were recruited over a 23-month period under protocols approved by local hospital Institutional Review Board on established inclusion (confirmed prostate cancer diagnosis, consented to adhere to FMD) and exclusion criteria (poorly controlled diabetes, or hypertension). 29 patients completed the study, and no patient was lost to follow up. Participants were monitored with baseline and evaluation consultations. There was an option for telephone consultation at any point for the patient if there were any issues regarding adherence or adverse effects. Any adverse effects were reported retrospectively by the patient (Fig. 1).

### Intervention

#### Experimental FMD

Participants were instructed to consume only the content of an FMD box, for 4 continuous days, and to return to their normal diet after completion until the next cycle. Participants completed 3 cycles of this 4-day FMD.

#### FMD

The FMD is a 4-day plant-based low amino-acid substitution diet. Calorie content declined from day 1 (~4600 kJ), to days 2–4 (~840kJ). Moreover, the carbohydrates/proteins/fats energy ratio was approximately 3.5/1/7on the first day, while complex carbohydrates were the main macronutrient (>80 energy%) with fats representing about 10% of energy consumption in the subsequent 3 days.

The FMD comprises proprietary formulations belonging to USC and L-Nutra of vegetable-based soups, energy bars, energy drinks, chip snacks, tea, and a supplement providing high levels of minerals, vitamins, and essential fatty acids. All items to be consumed per day were individually boxed to allow the subjects to choose when to eat while avoiding accidentally consuming components of the following day.

For the remainder of the time between cycles, patients were allowed to “eat as per their normal diet”.

#### Assessments

Participants were assessed by their clinician at baseline prior to starting the intervention, and at least 3 months after completing their last FMD cycle, to allow a “washout time” to assess the durability of the results.

The primary study assessments were body weight, abdominal circumference, blood pressure and secondarily, selected laboratory measurements.

#### Blood tests & serum markers

As this was a pilot study design, in a real-world community oncology setting, selected laboratory studies were sought from the patient’s GP, and recorded when available. These included: metabolic markers, random and fasting blood glucose, total cholesterol, HDL & LDL cholesterol, triglycerides, HbA1c, Prostate Specific Antigen (PSA), selected inflammatory markers which were available in a subset of patients (uric acid, CRP etc). (Note: The investigators planned to include these in a prospective fashion, but in this pragmatic setting, were only able to include those available on retrospective analysis.)

#### Statistical analysis

The data was imported into the R programming language (version 4.0.4) to facilitate advanced statistical analysis. Data analysis was supported by both descriptive and inferential analyses. In particular, descriptive statistics tables along with waterfall plots were presented to show a summary of information from each of the variables, while two-sided paired-sample *t*-test will be performed to assess the DMS’s effect for each variable. For variables with small sample size (i.e., *n* < 25), the Normality assumption was verified by looking at the boxplots while Central Limit Theorem was applied for variables with large sample size (i.e., *n* > 25). The results were evaluated at 95% confidence interval and *p* value <0.05 was accepted as statistically significant.

## Results

### Baseline data for all subjects

From January 2019 to November 2020, 35 study participants were enrolled in our observational study, 29 of whom completed the 3 cycles of FMD. 6 patients withdrew during the study due to difficulty in adhering to the FMD protocol. 3 of them withdrew during the 1st cycle of FMD and 3 participants withdrew during the 2nd cycle. Adherence was assessed via an in-person consultation 3 months post the 3rd and final FMD cycle. The most common reasons cited for failing to adhere were: coincidence with holidays (e.g., Christmas, Easter etc), taste preference around herbal teas, feeling of fatigue/weakness at times. There were no discernible medical events requiring physician intervention. All were reported by participant recall. There was no formal nutritional healthcare professional support available, which may have contributed to 5 of the 6 patients “failing to complete” the trial. Some of the dropouts for the reasons cited might have been avoided with more patient support during the study. However, one patient noted difficulty maintaining the “strict dietary” regime (Table 1).

**Table 1:** Characteristics of all subjects at enrollment.

Characteristics at baseline:	(*n* = 35)
*Sex: n (%)*	
Male	35 (100)
*Age (years)*	
Mean	69 ± 6.9
*Smoking status: n(%)*	*(n* = *16)*
Never	7 (44)
Previous smoker	9 (56)
*Combined Gleason score: n(%)*	*(n* = *16)*
6	3 (19)
7	6 (37.5)
8	6 (37.5)
9	1 (6)
*Prostate Cancer status: n(%)*	
Prostate Cancer (primary treatment)	26 (74)
Prostate Cancer (Relapsed)	9 (26)
*Treatment: n(%)*	
ADT	7 (20)
BXT^a^	7 (20)
Triple therapy^b^	3 (9)
ADT & BXT	3 (9)
ADT & EBRT^c^	5 (14)
Post Triple therapy	2 (6)
Post ADT and EBRT	6 (17)
Prostatectomy Radiotherapy & BXT	1 (3)
Post EBRT	1 (3)

Plus-minus values are means ± SD rounded to the nearest 10th.

^a^BXT is brachytherapy.

^b^Triple therapy is ADT, EBRT and brachytherapy.

^**c**^EBRT is external beam radiotherapy.

### Adverse effects & safety

No significant adverse effects were noted among our cohort. No re-feeding problems were reported.

### Comparison of risk factors and datapoints from baseline to evaluation

At evaluation (3 months after completing 3 consecutive rounds of the FMD) we assessed any changes in the risk factors and metabolic markers that we examined in the 29 patients that completed the FMD regime (Table 2).

**Table 2:** Risk factor/Metabolic changes in subjects who completed the trial.

Variable	Baseline *n*	Baseline mean ± SD	Evaluation *n*	Evaluation mean ± SD	Average difference	(95% CI)	Efficacy (*p* value)
Body weight (kg)	35	98 (17)	29	92 (15)	−3.8	(−5.61, −1.97)	0.0002
BMI (kg/m^2^)	32	32 (6.8)	29	30 (5.6)	−1.2	(−1.81, −0.67)	0.0001
Abdominal Circumference (cm)	34	111 (12)	29	105 (12)	−4.6	(−6.87, −2.27)	0.0003
Systolic Blood Pressure (mmHg)	35	138 (18)	29	128 (18)	−9.5	(−16.16, −2.88)	0.0066
Diastolic Blood Pressure (mmHg)	35	81 (12)	29	77 (11)	−4.5	(−8.54, −0.43)	0.0316
Triglycerides (mg/dL)	25	1.5 (0.67)	19	1.3 (0.46)	−0.22	(−0.52, 0.07)	0.127
Total Cholesterol (mg/dL)	26	4.6 (0.85)	20	4.5 (1.1)	−0.2	(−0.42, 0.01)	0.0634
LDL Cholesterol (mg/dL)	26	2.6 (0.65)	20	2.6 (0.97)	−0.13	(−0.37, 0.12)	0.2887
HDL Cholesterol (mg/dL)	26	1.3 (0.28)	19	1.4 (0.39)	0.07	(−0.02, 0.15)	0.1178
HbA1C	19	41 (8.8)	14	39 (11)	−4.4	(−8.97, 0.26)	0.0625
PSA (ng/dL)	26	2.3 (3.8)	20	3.7 (7.5)	0.39	(0.1, 0.68)	0.0112

*CI* confidence interval.

Several metabolic factors showed improvements post intervention, and some borderline responses. On average the weight of patients’ receiving 3 FMD cycles dropped by 3.79 kg after the 3 months, within which the true reduction is likely to be between 1.97 and 5.61 kg with 95% confidence. Abdominal circumference was reduced on average by 4.57 cm. The FMD cycles also resulted in a decrease in systolic BP with average difference of −9.52 mmHg. Diastolic BP was reduced by 4.48 mmHg.

**Fig. 2:**
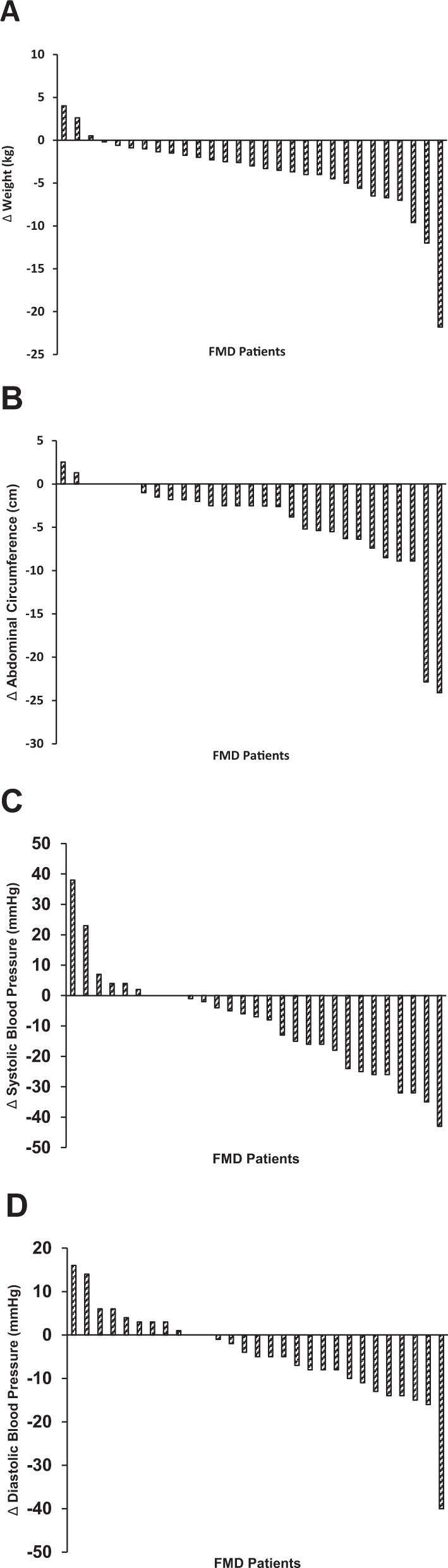
Waterfall plots showing changes in risk factors & metabolic markers from baseline to evaluation for each patient (*n* = 35). Results of metabolic syndrome risk factors and metabolic markers in all subjects who completed the FMD regime. **A** Body weight, **B** Abdominal circumference, **C** Systolic Blood Pressure, **D** Diastolic Blood Pressure. All data represents average difference between baseline data and evaluation (i.e., weight at evaluation minus weight at baseline) of individual FMD participants. For some of the enrolled participants our team were unable to collect laboratory samples from all subjects. We therefore excluded subjects with incomplete measurements from a particular marker group as seen in Table 2. ∆, difference in datapoint from evaluation to baseline. **A**–**D** Change analysis of metabolic variables from baseline to evaluation.

In summary, three cycles of the FMD improved important metabolic risk factors in these patients, including reduced body weight, abdominal circumference, and blood pressure (Fig. 2, Tables 2 and 3).

**Table 3:** Sub-analysis of risk factor/metabolic changes in subjects who completed the trial.

Variable	*n*	Average Difference	(95% CI)	*p* value
Triglycerides < 1.7 mmol/L	12	0.07	(−0.20, 0.34)	0.5711
Triglycerides > 1.7 mmol/L	7	−0.73	(−1.25, −0.21)	0.0141
Total Cholesterol < 5.2 mmol/L	15	−0.27	(−0.52, −0.01)	0.0453
Total Cholesterol ≥ 5.2 mmol/L	5	−0.01	(−0.53, 0.50)	0.9516
LDL Cholesterol < 3.4 mmol/L	18	−0.14	(−0.41, 0.13)	0.2821
LDL Cholesterol ≥ 3.4 mmol/L	2	0.02	(−0.23, 0.27)	0.5
HDL Cholesterol ≥ 1 mmol/L	17	0.06	(−0.034, 0.16)	0.1796
HDL Cholesterol < 1 mmol/L	2	0.09	(−0.04, 0.22)	0.07
HbA1C < 42 mmol/L	8	−4.12	(−12.74, 4.50)	0.2958
HbA1C ≥ 42 mmol/L	6	−4.67	(−9.00, −0.33)	0.0395
BMI < 30	16	−1.05	(−1.73, −0.73)	0.005
BMI ≥ 30	13	−1.47	(−2.53, −0,41)	0.0105
Abdominal circ. ≥ 102 cm	21	−5.07	(−8.15, −0.98)	0.0026
Abdominal circ. < 102 cm	8	−3.28	(−6.20, −035)	0.0328
Systolic BP ≥ 130 mmHg	24	−16.04	(−23.26, −8.82)	0.0001
Systolic BP < 130 mmHg	9	−0.78	(−14.09, 12.53)	0.89
Diastolic BP ≥ 85 mmHg	11	−9.55	(−17.86, −1.23)	0.0028
Diastolic BP < 85 mmHg	18	−1.39	(−5.56, −2.79)	0.49

*CI* confidence interval.

To further evaluate the efficacy of FMD on metabolic risk factors in patients with prostate cancer, we performed a sub-analysis comparing the changes in stratified subgroups by risk factor, as defined by the baseline levels of each risk factors.

We selected clinically relevant cut-offs for metabolic syndrome and compared normal and at-risk subjects for each risk factor: abdominal obesity identified by waist circumference ≥102 cm in men, triglycerides levels ≥1.7 mmol/l, systolic BP ≥ 130 mmHg and HDL cholesterol <1 mmol/l, are all associated with an increased risk for cardiovascular disease; as well as an impaired glucose metabolism here identified by a Hb1Ac ≥ 42 mmol/l, indicating the presence of prediabetes or diabetes.

We observed a significant benefit of the FMD on BMI and abdominal circumference in all subgroups (*p* value <0.05), although the FMD had more impact in reducing abdominal obesity among subjects who were obese at baseline (BMI ≥ 30).

HbA1c was reduced more in patients with baseline levels ≥42 mmol/L; triglycerides were reduced significantly more in patients with baseline levels higher than 1.7 mmol/l.

Subjects with baseline levels of systolic BP ≥ 130 mmHg experienced a greater reduction in BP by the end of the FMD than those with baseline systolic BP < 130 mmHg. Diastolic BP did not change for patients with baseline levels <85 mmHg but was reduced by FMD in subjects with diastolic BP ≥ 85 mmHg.

Total cholesterol was reduced significantly in patients with baseline levels <5.2 mmol/l but not in those with baseline levels ≥5.2 mmol/l. FMD did not reduce HDL cholesterol in participants with levels above or below 1 mmol/l.

These results indicate that FMD cycles have stronger effects in at-risk patients than those with normal values of risk factors for metabolic syndrome.

## Discussion

The importance of disorders in body weight and metabolic health are increasingly acknowledged in the prevention and management of prostate cancer. Increased waist circumference is associated with an increased risk of prostate cancer, especially aggressive prostate cancer [11]. Elevated BP is associated with increased risk of death from prostate cancer [12]. Chronic illnesses such as type II diabetes and hypertension often coexist with PC in our clinics. Patients not infrequently report reductions in quality of life related to these metabolic factors. A large proportion of men require ADT as a component of therapy, some lifelong. Many of these men develop features of the metabolic syndrome, and the case has been made that while vital in optimizing cause-specific mortality in relapsed PC, ADT itself might contribute to an overall increase in mortality attributed to increased cardiovascular and other deaths, possibly offsetting the cause-specific benefits [13]. Patients frequently seek nutritional advice in oncology clinics, yet there is no standard recommendation for nutritional intervention in this setting [14]. Pharmacologic interventions are now being tested, including those with metformin, an anti-hyperglycaemic medication. One recent study has shown that metformin is non-toxic in this subset of patients but has yet to be proven beneficial [15].

Fasting in various forms has been shown highly effective in the management of several chronic medical illnesses such as type II diabetes and hypertension. However, recommendations to ‘fast’ in cancer therapy have been limited, possibly because of safety concerns. Prior work for one of our investigators (VL) has demonstrated the use of both fasting and FMDs in cancer treatment in both mouse and human studies. Fasting/FMDs have been used to mitigate the toxicity of chemotherapy, and to improve metabolic risk factors in a randomized control setting involving normal volunteers [10].

We therefore chose to test this approach in a small prospective opportunistic trial involving a cohort of men with prostate cancer, and we believe this study represents the first report on the use of FMD cycles in PC patients. Two major features of metabolic syndrome were reduced by implementing an FMD in this cohort, indicating that FMD cycles could have a role in the supportive management of a subset of patients with prostate cancer, especially those receiving ADT with metabolic impairment. The degree of metabolic impairment varied considerably across the cohort. The treating clinicians observed however, the more severe the degree of metabolic impairment, the better the response to the FMD, as was reflected in our sub-analysis (Table 3).

FMDs were developed to promote the effects of fasting while standardizing dietary composition, providing nourishment, and minimizing the burden and side effects associated with water-only fasting [16]. Fasting results in nutritional ketosis which promotes potent changes in metabolic pathways, which may result in cancer growth reduction, not testable in this study setting.

We believe the study showed important improvements signals in metabolic risk factors (body weight, abdominal girth, BP), potentially relevant to the overall outcome of patients with PC, especially those with significant metabolic impairment from ADT. The majority of the subjects reported the program was easy to undertake resulting in an 83% compliance to 3 cycles, a 91% compliance to 2 cycles, and a 100% compliance to 1 cycle. Importantly, no side effects attributable to the FMD were seen. The (17%) withdrawal of 6 participants from our study including some dropouts due to the difficulty in adhering to the strict FMD protocol, are consistent with other reports, e.g., 23% withdrawal from a larger cohort of patients undergoing intermittent fasting [17].

The ability of the FMD to result in effects on metabolic markers lasting over 3 months after its completion, underlines the potential importance of compliance to even 1 cycle. Therefore, even patients who cannot complete 3 consecutive FMD cycles but who could complete a lesser number, could benefit, a possibility which should be tested in future clinical studies.

There are a number of clear limitations to this study. These include the small cohort size, the heterogeneity of the patients in terms of stage and extent of disease, as well as the phase of their treatment. For example, some were in the midst of active treatment (primary as well as salvage post relapse) including ADT, and some had completed their therapies. Study participants were instructed not to change their normal diet and lifestyle for the duration of the study (excluding days of FMD regime), but some reported making voluntary changes once they saw the benefits of these interventions and better understood the importance of food choice in the context of their illness. We cannot therefore be sure the observed benefits were strictly down to the intervention alone, and further studies would be required to evaluate this possibility, ultimately involving a randomized control arm design.

Overall, we believe FMD is safe, shows promise in improving metabolic health in cancer patients, and may deserve further study in PC.

## Data Availability

The datasets generated and analyzed during the current study are not publicly available as they are part of the confidential medical record but are available from the corresponding author on reasonable request.
