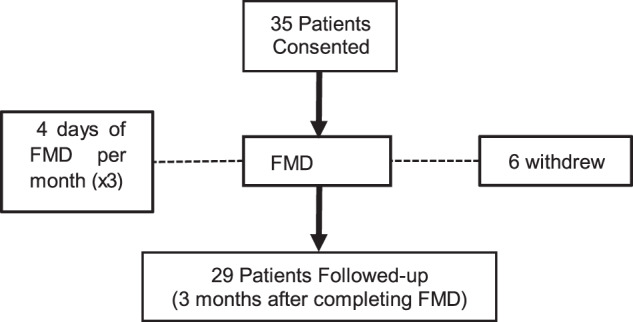# Correction: The impact of a fasting mimicking diet on the metabolic health of a prospective cohort of patients with prostate cancer: a pilot implementation study

**DOI:** 10.1038/s41391-022-00540-7

**Published:** 2022-05-18

**Authors:** V. Fay-Watt, S. O’Connor, D. Roshan, A. C. Romeo, V. D. Longo, F. J. Sullivan

**Affiliations:** 1 School of Medicine, National University of Ireland, Galway, Ireland; 2 Department of Radiation Oncology, Galway Clinic, Doughiske, Galway, Ireland; 3 School of Mathematical and Statistical Sciences, National University of Ireland, Galway, Ireland; 4 CURAM, SFI Research Centre for Medical Devices, National University of Ireland, Galway, Ireland; 5 Department of Internal Medicine and Medical Specialties, IRCCS Ospedale Policlinico San Martino, Genova, Italy; 6 IFOM, FIRC Institute of Molecular Oncology, Via Adamello 16, 20139 Milano, Italy; 7 Longevity Institute, School of Gerontology, Department of Biological Sciences, University of Southern California, 3715 McClintock Avenue, Los Angeles, CA 90089-0191 USA; 8 Department of Radiation Oncology, Galway Clinic, Prostate Cancer Institute, National University of Ireland, Galway, Ireland

**Keywords:** Cancer therapy, Cancer prevention, Prostate cancer, Prostate cancer, Cancer metabolism

Correction to: *Prostate Cancer and Prostatic Diseases* 10.1038/s41391-022-00528-3, published online 21 March 2022

In this article the wrong figure appeared as Fig. 1; the figure should have appeared as shown below. The original article has been corrected.